# The Challenge of the Sponge *Suberites domuncula* (Olivi, 1792) in the Presence of a Symbiotic Bacterium and a Pathogen Bacterium

**DOI:** 10.3390/genes10070485

**Published:** 2019-06-26

**Authors:** Gaël Le Pennec, Johan Gardères

**Affiliations:** Laboratoire de Biotechnologie et de Chimie Marines, Université de Bretagne Sud, EA 3884–IUEM, 56100 Lorient, France

**Keywords:** apoptosis, immune response, *Suberites domuncula*, *Endozoicomonas* sp., *Pseudoalteromonas* sp.

## Abstract

Sponges, which are in close contact with numerous bacteria in prey/predator, symbiotic and pathogenic relationships, must provide an appropriate response in such situations. This starts with a discriminating recognition of the partner either by a physical contact or through secreted molecules or both. We investigated the expression of the Toll-like receptor, Caspase 3/7, Tumor Necrosis Factor receptor-associated factor 6, Bcl-2 homology protein-2 and macrophage expressed genes of axenic sponge cells in the presence of a symbiotic bacterium (*Endozoicomonas* sp. Hex311), a pathogen bacterium (*Pseudoalteromonas* sp. 1A1), their exoproducts and lipopolysaccharides. The vast majority of answers are in line with what could be observed with the symbiotic bacterium. The pathogenic bacterium seems to profit from the eukaryotic cell: suppression of the production of the antibacterial compound, inhibition of the apoptosis caspase-dependent pathway, deregulation of bacterial recognition. This work contributes new scientific knowledge in the field of immunology and apoptosis in early branching metazoan harboring within its tissue and cells a large number of symbiotic bacteria.

## 1. Introduction

Sponges and bacteria co-evolved for millions years, leading to stable and long-lasting associations. According to their microbial composition within their tissues, including bacteria, archaea, fungi and microalgae, sponges were recently divided into two groups: the high microbial abundance (HMA) and low microbial abundance (LMA) sponges [1]. These microbial communities can reach up to 35–40% of the biomass of the animal in some species [2,3], or up to about 10^9^ microbial cells/cm^3^ [4], which represent 47 phyla of bacteria within the same sponge [5]. These arguments allow classifying these superorganisms as holobionts according to the definition of Zilber-Rosenberg and Rosenberg [6]. A focus on such an association could shed light on host-bacteria interactions in basal metazoans particularly providing information on how the host discriminates between a symbiotic versus a pathogenic bacterium and how it addresses this ongoing presence with appropriate molecular mechanisms.

Indications regarding an intricate network of interactions including a shared metabolism emerged progressively in the Poriferan world [7]. Nevertheless, communications and interactions between both partners remain poorly explored and understood [8,9]. Sponge-associated bacteria may interact with their host by a direct physical interaction, a molecular dialogue or both [8]. In the demosponge *Suberites domuncula* (Heteroscleromorpha, Suberitidae), a first step in physical communication was well illustrated by the recognition of pathogen-associated molecular patterns (PAMP). Indeed, lipopolysaccharides (LPS), lipopeptides and proteoglycans stimulate the sponge innate immune response through the toll/interleukin-like receptor (TLR/ILR), the *Suberites domuncula* LPS interacting protein (SDLIP), the myeloid differentiation primary response 88 (MyD88), the interleukin receptor-associated factor (IRAK) and the Tumor Necrosis Factor receptor-associated factor 6 (TRAF-6) [10,11]. LPS from *Escherichia coli* increased the expression of several antibacterial effectors from *S. domuncula* including the macrophage expressed gene (MPEG) [11]. The lipopeptide Pam(3)Cys-Ser-(Lys)(4) stimulated the expression of the *S. domuncula* caspase 3/7-like [10] and proteoglycans increased the transcription of the lysozyme gene in this animal [12]. More recently, it was postulated that bacteria could also interact with some sponge membrane proteins through eukaryotic protein-like domains, including ankyrin domains, tetracopeptide-like domains and fibronectin domains, borne by members of the symbiont candidate phylum Poribacteria. These interactions would contribute to the recognition of sponge-associated microorganisms [13,14].

Sponge-associated bacteria also interacted with their host through secreted molecules. Stimulation of sponge cell cultures with *N*-3-oxo-dodecanoyl-L-homoserine lactone (3-oxo-C_12_-HSL), a bacterial quorum sensing molecule produced within the sponge *S. domuncula*, led to a decrease of the host immune and apoptotic responses and, conversely, to an increase of phagocytosis-related genes expression [8,9]. A lectin isolated from the sponge *Halichondria panicea* also stimulated the in vitro proliferation of the sponge-associated *Pseudomonas insolita*, whereas it did not influence the growth of the other sponge-isolated bacteria [15]. Gardères et al. [16] reviewed the roles of porifera lectins and their potential involvement in the association between sponges and microorganisms.

In a previous work, we isolated an opportunistic pathogenic bacterium (*Pseudoalteromonas* sp. 1A1) and a commensal, potentially mutual, bacterium (*Endozoicomonas* sp. Hex311) from the low microbial abundance sponge *S. domuncula* [9]. LPS from both strains were characterized and their effects on axenic sponge 3D cell cultures were compared to those of *E. coli* regarding the regulation of the MPEG gene [17]. In the present study, we investigated the effect of LPS and culture supernatants from both bacteria regarding immune and apoptotic pathways genes in order to have a wider comprehension of the role of these two bacteria on their host. In the meantime, the effects of co-incubation of axenic sponge cell-cultures with both bacterial strains at a macroscopic level were studied.

## 2. Materials and Methods

### 2.1. Specimen Collection and Ethical Statement

Sponges used for the following experiments were collected by scuba diving at 0–10 m depth in Roscoff (48°68′64.78′′N, 3°94′18.65′′W), Britany, France. Specimens were immediately transferred into aquariums under controlled conditions of temperature (12 °C) and luminosity (10–14h light/dark) in a closed circulating system of natural seawater. Sponges were fed twice a week with a protein solution according to Le Pennec et al. [18]. No specific authorization was required to collect sponges in open areas for scientific purposes. This study did not involve endangered or protected species. Protocols used in this work were in accordance with Federation of European Laboratory Animal Science Association guidelines and the National Law for Laboratory Animal Experimentation (Law No. 18.611).

### 2.2. Bacterial Cultures

*Endozoicomonas* sp. Hex311 and *Pseudoalteromonas* sp. 1A1 were cultivated from a stock solution stored at –80 °C in LB/glycerol medium (75/25). For LPS extraction, bacteria were grown according to Gardères et al. [17] in a Zobell medium (1.30 g·L^−1^ yeast extract and 6.61 g·L^−1^ peptone in sea water, pH 7.4) supplemented or not with sponge extract according to the strain needs. Before collecting the supernatant, bacteria were cultivated at 20 °C. Two hours after the beginning of their respective stationary phase a centrifugation at 3000× *g* for 10 min at room temperature (RT, 20 °C) separated the culture supernatant from cells. Supernatants were then 0.2 µm filtered to remove any remaining bacteria or debris. Pellets were suspended in the same volume of fresh Zobell medium prior to an incubation with sponge primmorphs during co-culture experiments.

### 2.3. Lipopolysaccharides Preparation

*Escherichia coli* 0111:B4 lipopolysaccharides were purchased from Sigma-Aldrich (St. Quentin Fallavier, France). LPS preparations from *Endozoicomonas* sp. Hex311 and *Pseudoalteromonas* sp. 1A1 were performed according to Westphal and Jann [19]. Briefly, 100 mL of a bacterial culture collected 2 h after the beginning of the stationary growth phase were centrifuged at 3000× *g* for 10 min at RT. The pellet was suspended in distillated water (17 mL per g of bacteria) and heated at 65 °C. An equal volume of phenol 90% (v/v) preheated at 65 °C was added to the bacterial suspension. The solution was vigorously mixed for 1 min and further incubated at 65 °C, for 15 min, under permanent agitation. After incubation onto an ice bath for 10 min, the solution was centrifuged at 3000× *g* for 45 min, at 4 °C. The upper phase was stored at 4 °C and the lower phase was extracted once again with an equal volume of distillated water at 65 °C with the same procedure. Both aqueous phases were gathered together and dialyzed against distilled water for three days (membrane Molecular Weight Cut Off 4000–6000 Da) to remove remaining molecules of phenol. The solution was then centrifuged at 3000× *g* for 45 min, at 4 °C. The supernatant was frozen at –80 °C before freeze-dried. Dried extract was suspended in 3% (w/v) of distillated water and then centrifuged at 80,000× *g* for 6 h, at 4 °C. The pellet was suspended in one milliliter of distillated water and then centrifuged once again at 105,000× *g* for 3 h, at 4 °C. The final pellet was then suspended in distillated water at a final concentration of 2 mg·mL^−1^.

### 2.4. Sponge Cell Dissociation and Primmorphs Culture

Sponge cells were individually isolated from three specimens of *S. domuncula* as described by Le Pennec et al. [18]. The monitoring of bacteria and primmorphs interactions was performed in triplicate in 96-wells plates. Sponge dissociated cells (10^6^ cells·mL^−1^) were first cultivated for 2 days in seawater supplemented by sea silica-enriched sand (Sigma-Aldrich, St. Quentin Fallavier, France), 10 mM ferrous citrate (Merck Chimie SAS, Fontenay sous bois, France), 1 mM of pyruvate (Sigma-Aldrich, St. Quentin Fallavier, France), 73.0 mg·mL^−1^ of Penicillin G potassium and 34.5 mg·mL^−1^ of Streptomycin sulfate to prevent any bacterial contamination. Subsequently, they were contaminated by alive bacterial cells, either *Endozoicomonas* sp. Hex311 or *Pseudoalteromonas* sp. 1A1, with a final optical density at 600 nm (OD_600nm_) of 0.1 in the same culture medium as mentioned above but without any antibiotic. An equal volume of Zobell medium was added in control cultures. The plates were incubated at 15 °C with a gentle agitation. The bacterial growth was monitored at the end of experiments by measuring the OD_600nm_. Data were statistically compared using the software Statgraphics^®^ Centurion VII (Statgraphics Technologies, Inc., The Plains, VA, USA).

Regarding gene expression analysis, primmorphs were prepared in the same manner as previously described [17] and co-incubated at 15 °C for 16 h with alive *Endozoicomonas* sp. Hex311 or *Pseudoalteromonas* sp. 1A1 strains with an initial OD_600nm_ of 0.1 under a gentle shaking, the same as the one for primmorphs culture [18]. Primmorphs culture stimulations were also performed in the presence of 1 mL/20 mL of culture medium of 0.2 µm-filtered *Endozoicomonas* sp. Hex311 or *Pseudoalteromonas* sp. 1A1 culture supernatants in the same conditions. In both cases, a control culture was realized in parallel with 1 mL of sterile Zobell medium.

### 2.5. Quantitative Reverse Transcription (qRT-PCR) Analyses

Total RNAs were isolated from primmorphs using the extraction solution RNA now (Epicentre, Madison, WI, USA) and DNase treated (Epicentre, Madison, MI, USA) according to the manufacturer’s instructions. A total of 500 ng of total RNAs were used to synthesize first-strand cDNAs using oligodT primers with MultiScribe Reverse Transcriptase (Applied Biosystem, Foster City, CA, USA) according to the manufacturer’s instructions. A negative control was included using total RNAs without the MultiScribe Reverse Transcriptase. mRNAs of interest were quantified by real-time PCR amplification of their cDNAs. The relative mRNA transcript changes in connection with every treatment were studied for the caspase-like 3/7, BCl_2_-homolog protein (SDBHP-2), the Toll-like receptor (TLR), the TNF receptor associated factor 6-like protein (TRAF-6) or the macrophage expressed gene (MPEG) genes, using the genes Hypoxanthine-guanine phosphoribosyltransferase (HPRT) and glyceraldehyde-3-phosphate dehydrogenase (GAPDH) as housekeeping genes [9]. PCR reactions were performed in triplicate with the 7300 Real Time PCR System apparatus (Applied Biosystems, Foster City, CA, USA). A total of 20 mL of reactions contained 10 μL SYBR Green PCR Master Mix (including AmpliTaq Gold DNA Polymerase) (Applied Biosystems, Foster City, CA, USA), 2 μL of each primer (3 mM) and 8 μL of cDNAs. For each experiment, three serial cDNA dilutions were made: 250.0, 25.0 and 2.5 pg·mL^−1^. qPCR conditions were 95 °C for 10 min for polymerase activation, and 40 cycles at 95 °C and 60 °C for 60 s and 30 s, respectively. The relative quantification of the mRNAs of interest was obtained by the comparative Pfaffl method [20] using HPRT or GAPDH as endogenous control [9]:

Ratio (R) = E _sample gene_^(Ct control−Ct stimulated)^/E _reference gene_^(Ct control−Ct stimulated)^

where E is the efficiency of the primer couple, and Ct is the cycle threshold. Relative mRNA level values resulted from calculated values: Values above and below 1 showed a higher and a lower mRNA level in the presence of a treatment, respectively (e.g., a value of 1.5 indicated a 1.5-fold increased mRNA level in stimulated condition; and a value of 0.66 revealed that the mRNA level was divided by 1.5 in stimulated condition). An ANOVA test was performed as a statistical test with the following hypothesis: the results are different between the controls and the samples or between two samples (*p* < 0.05) using the software Statgraphics^®^ Centurion VII.

## 3. Results

### 3.1. Interactions between Sponge-Isolated Bacteria and Primmorphs Cultures: Impact at Macroscopic and Molecular Levels

**Figure 1:**
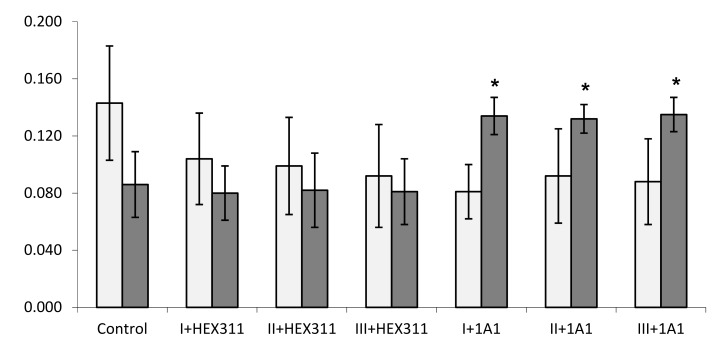
Evolution of *Endozoicomonas* sp. Hex311 (HEX311) and *Pseudoalteromonas* sp. 1A1 (1A1) co-cultivated in presence of three batches (I, II, and III) of *Suberites domuncula* primmorphs during 16 h. The growth status of both bacteria was estimated by measuring the OD_600nm_ in the supernatant of cell cultures at the end of the experiment. (*) Statistically significant differences of growth (*p* < 0.05) are pointed out by an asterisk. 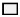 OD_600nm_ after inoculation of bacteria at T0; 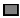 OD_600nm_ after 16h of co-culture.

The growth evolution of *Endozoicomonas* sp. Hex311 and *Pseudoalteromonas* sp. 1A1 in the presence of primmorphs was evaluated by following the OD_600nm_ after 16h of co-incubation (Figure 1). The OD_600nm_ remained stable for *Endozoicomonas* sp. Hex311 whereas it increased for *Pseudoalteromonas* sp. 1A1, reflecting a statistically significant growth only for the latest bacterium. In the absence of sponge primmorphs none of both bacteria incubated grew.

### 3.2. Co-cultivation of Primmorphs and Alive Bacteria

**Figure 2:**
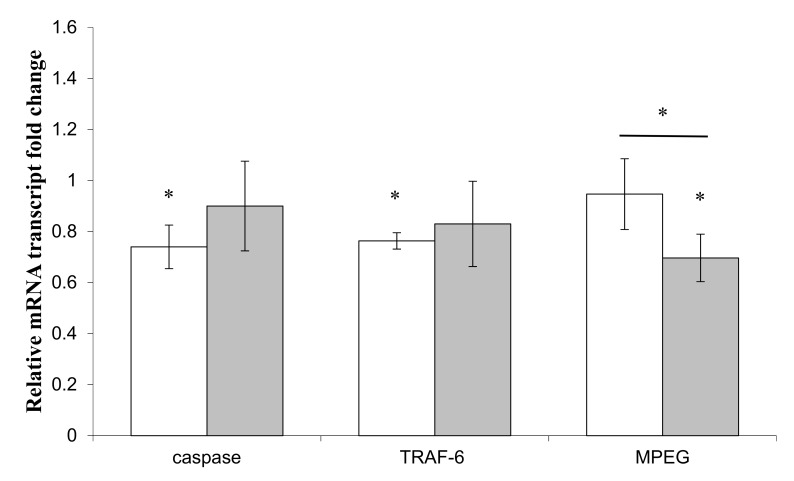
Expression of the macrophage expressed gene (MPEG), Tumor Necrosis Factor receptor associated factor 6-like protein (TRAF-6) and caspase genes in primmorphs co-cultivated in the presence of alive sponge-isolated bacteria; *Endozoicomonas* sp. Hex311 (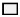) or *Pseudolateromonas* sp. 1A1 (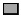). (*) Statistical differences between the controlled and the stimulated condition are materialized by an asterisk and statistical differences between primmorphs stimulated by the two bacteria are materialized by a horizontal bar.

Sponge primmorphs were co-incubated for 16 h in the presence of alive sponge-isolated bacteria, either *Pseudoalteromonas* sp.1A1 or *Endozoicomonas* sp. Hex311, in order to study their impact on the expression of two immune (TRAF-6 and MPEG) and one apoptotic (caspase-like 3/7) genes (Figure 2). The caspase and TRAF-6 genes expression presented a significant decreased (average ratio of 1.4) in the presence of *Endozoicomonas* sp. Hex311, compared to the control cultures; *Pseudoalteromonas* sp. 1A1 did not affect these expressions. *Pseudoalteromonas* sp. 1A1 statistically down-regulated the expression of the MPEG gene compared to the control conditions (average ratio of 1.5). The comparison of primmorphs genes expression in the presence of both bacteria did not statistically evidence any difference except for the MPEG gene.

### 3.3. Incubation of Primmorphs in the Presence of Bacterial Culture Supernatant

**Figure 3:**
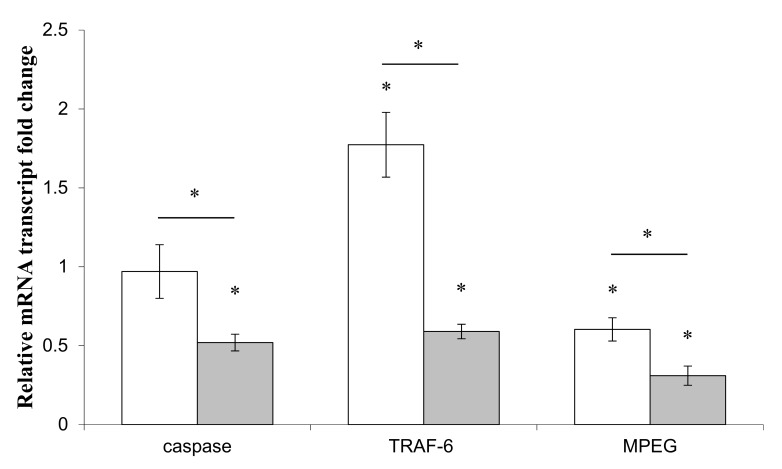
Expression of genes related to the immune system, MPEG and TRAF-6 and to the apoptotic pathway, caspase, in primmorphs co-incubated in the presence of 1mL/20 mL of supernatant of the sponge-isolated bacterium *Endozoicomonas* sp. Hex311 (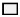) or *Pseudolateromonas* sp. 1A1 (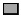), respectively. (*) Statistical differences between the controlled and the stimulated condition are materialized by an asterisk and statistical differences between primmorphs stimulated by the two bacteria are materialized by a horizontal bar.

Primmorphs were incubated in the presence of the supernatants of *Endozoicomonas* sp. Hex311 or *Pseudoalteromonas* sp. 1A1 in order to investigate the impact of bacterial secreted molecules on two genes of the sponge immune system, TRAF-6 and MPEG, and one gene of the sponge apoptotic system, the caspase-like 3/7 (Figure 3). The expression of the caspase-like 3/7 was revealed to be statistically different in the presence of both supernatants. The expression of caspase did not change in the presence of *Endozoicomonas* sp. Hex311 supernatant, whereas it significantly decreased with an average factor of 2 in the presence of *Pseudoalteromonas* sp. 1A1 supernatant, compared to control cultures. The expression of TRAF-6 was also statistically different in both cases. The *Endozoicomonas* sp. Hex311 supernatant increased significantly the expression of TRAF-6 with an average factor of 1.8 and, conversely, *Pseudoalteromonas* sp. 1A1 decreased the expression of the gene with an average factor of 1.7, compared to control cultures. Both supernatants statistically decreased the expression of the MPEG gene with an average ratio of 1.7 for *Endozoicomonas* sp. Hex311 supernatant and 3.2 for *Pseudoalteromonas* sp. 1A1 supernatant. This decrease was revealed to be statistically higher in the presence of *Pseudoalteromonas* sp. 1A1 supernatant.

The presence of homoserine lactones in both supernatants was investigated by on-line high-performance liquid chromatography-mass spectrometric detection following the method of [21]. It did not show evidence of any of these molecules (data not shown).

### 3.4. Effects of LPS on Immune and Apoptotic Genes

**Figure 4:**
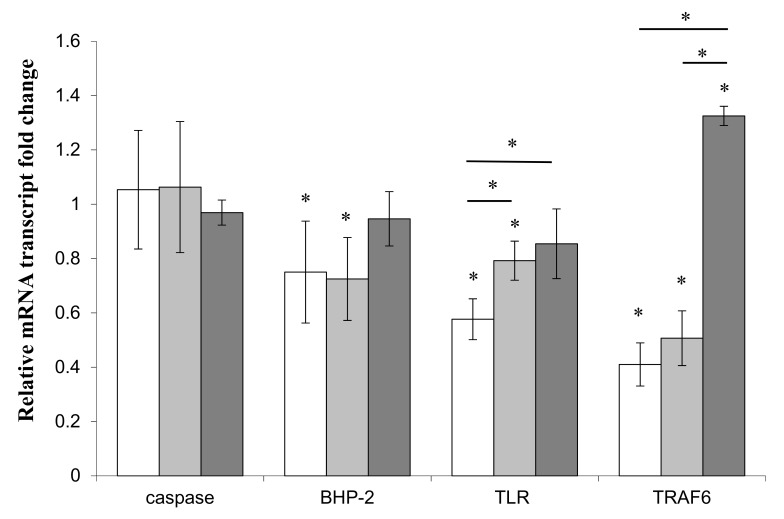
Expression of genes related to the immune system (Toll-like receptor (TLR) and TRAF-6) and to the apoptotic pathway (caspase and BCl_2_-homolog protein (BHP-2)) in primmorphs co-incubated in the presence of 2mg/mL of lipopolysaccharides of respectively the sponge-isolated bacterium *Endozoicomonas* sp. Hex311 (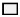) or *Pseudoalteromonas* sp. 1A1 (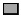) and the control bacterium *Escherichia coli* (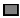). (*) Statistical differences between the standard and the stimulated condition a materialized by an asterisk and statistical differences between primmorphs stimulated by the two bacteria are materialized by a horizontal bar.

In order to complete the investigation on the effects of LPS from both *Endozoicomonas* sp. Hex311 and *Pseudoalteromonas* sp. 1A1 (cf. [17]), the expression of two apoptotic system genes, the caspase-like 3/7 and SDBHP-2, and two immune system genes, TLR and TRAF-6, was studied in the presence of LPS from both strains. *E. coli* LPS were used as reference (Figure 4). The expression of the caspase-like 3/7 did not present any variation in the presence of the three different LPS, whereas the expression of the SDBHP-2 gene significantly decreased in the presence of LPS from *Pseudoalteromonas* sp. 1A1 and *Endozoicomonas* sp. Hex311, with an average factor of 1.3 and 1.4, respectively, compared to the control culture without LPS. LPS from *E. coli* did not change the expression of SDBHP-2. Regarding the expression of the immune genes TLR and TRAF-6, their expression was modified in the presence of LPS. The TLR gene transcription significantly decreased in the presence of LPS from *Endozoicomonas* sp. Hex311 and *Pseudoalteromonas* sp. 1A1 (average factor of 1.8 and 1.2, respectively), but no change was observed for a stimulation with *E. coli* LPS. The expression of TLR gene was significantly lower in *Endozoicomonas* sp. Hex311 LPS-treated samples than in *Pseudoalteromonas* sp. 1A1 and *E. coli* LPS-treated samples. TRAF-6 gene expression was statistically modified either after treatment with LPS from *Endozoicomonas* sp. Hex311 and *Pseudoalteromonas* sp. 1A1 or with LPS from *E. coli*. The expression of TRAF-6 was statistically increased in the presence of LPS from *E. coli* with an average factor of 1.3, compared to the control culture, whereas its expression decreased significantly in the same manner in the presence of LPS from both *Endozoicomonas* sp. Hex311 and *Pseudoalteromonas* sp. 1A1 with average factors of 2.5 and 2, respectively.

## 4. Discussion

### 4.1. TLR

Toll-like receptors (TLRs) are members of the pattern recognition receptors (PRRs). They have a transmembrane localization and are dedicated to the recognition of a broad range of molecules produced by pathogenic organisms known as pathogen-associated molecular patterns or PAMPs and to the initiation of intracellular signaling [22]. For example, LPS as PAMPs activate TLRs [23]. TLRs play a crucial role in innate immunity [24] even in non-mammal organisms [25]. Concerning sponges, Wiens et al. [11] identified a TLR in *S. domuncula* (SDTLR), which shares homologies with the human TLR-1 and recognizing lipopeptides, like Gauthier et al. [26] for *Amphimedon queenslandica*. Due to its localization at the interfaces between the environment and the cell, SDTLR takes part in the antibacterial response of sponges [10,11]. In Porifera, it could also take part in the recognition of all the concerned bacterial genera including the symbiotic ones and guide the response according to the pathogenicity potential of the bacterium by modulating the immune response. Moreover, by extension, PRRs should perhaps be considered in the recognition of bacterial symbiotic-associated molecular patterns (SAMPs) in the forecasting of transient or perennial associations as well as pathogens. The arguments in this direction are scarce but exist [27,28].

Our comparison of primmorphs incubations in the presence of LPS from *E. coli*, *Endozoicomonas* sp. Hex311 and *Pseudoalteromonas* sp. 1A1 indicated a massive power of discrimination between the LPS from different bacteria. *Pseudoalteromonas* sp. 1A1 was responsible of a pathogenic event in our sponge culture tanks and *Endozoicomonas* sp. Hex311 was exclusively isolated and cultivated on sponge extracts supplemented Zobell medium. This may explain the differential responses we observed; that is, LPS from *Endozoicomonas* sp. Hex311 were the less immunostimulant whereas the ones from *E. coli* and *Pseudoalteromonas* sp. 1A1 were the most. Note that *E. coli* served as food supply for sponge experimental farming for a long time [28,29,30,31]. It seemed that there was no difference for the sponge *S. domuncula* between bacteria with nutritional and pathological fate, or this one was not made at the TLR level. Furthermore, *S. domuncula* was perfectly able to differentiate a symbiotic bacterium from the others. This is not so surprising since those animals co-evolved for many hundreds of millions years. The role of TLRs assumed in recognition of microbial invaders and leading to a recruitment of the innate immune system elements is then confirmed (see [11]). In our work, we demonstrated the role of TLR in recognition of symbiotic bacteria without presuming of a stimulation of the immune system. Insofar, SDTLR as a member of the PRRs is able to discriminate between bacterial PAMPs and also between PAMPs and SAMPs.

### 4.2. BHP-2

The Bcl-2 family is composed of pro- and anti-apoptotic molecules activated by extrinsic and intrinsic apoptotic signals [32] including those transmitted by TLRs [33]. Sponge Bcl-2 homologous Protein (BHP) has a high similarity to some mammalian Bcl-2 members and some of these molecules confer resistance against induction of apoptosis. *S. domuncula* BHP-2 (SDBHP-2) has an anti-apoptotic role as demonstrated by Wiens et al. [34,35].

Our results indicated that SDBHP-2 expression was down-regulated in the presence of LPS from *Endozoicomonas* sp. Hex311 and *Pseudoalteromonas* sp. 1A1 although LPS from *E. coli* did not affect its expression. The decrease of expression of SDBHP-2 in the presence of LPS from this symbiotic versus pathogenic bacterium gives the idea that the apoptotic activity was lowered. While it is perfectly understandable for the symbiotic bacterium, the question may arise for the pathogen one. Does it take the control of the apoptotic pathway in order to prevent sponge cells to commit suicide for its own benefit? This is an issue that cannot be resolved at this time with current knowledge. *E. coli* presence is highly limited within sponges to circulating seawater reflecting an environmental contamination by coliforms. Furthermore, when mentioned in literature it is as food supply for sponges [29,30,31,35,36]. Insofar, it is not surprising that this bacterium does not affect somehow or other the apoptosis. However, our results reflect the fact that this first Eumetazoa [37] is already perfectly capable of differentiating between bacteria on which it feeds from symbiotic and pathogenic bacteria and to provide a specific response.

### 4.3. Caspase

Caspases, as actors of the apoptosis, play a crucial role in the execution phase of cell death [38]. The *S. domuncula* caspase 3/7 shares homologies with caspases of higher vertebrates [11]. We previously described the influence of the bacterial molecule of communication 3-oxo-C_12_ HSL on the gene expression of this caspase [9]. This molecule down-regulated the expression of the caspase gene as we observed extensively herein in the presence of the culture supernatant of the bacterium *Pseudoalteromonas* sp. 1A1. One or several secreted molecules or extracellular vesicle (ECVs) [39] would be probably able to down-regulate or to inhibit the apoptosis pathway. LPS from this bacterium or the alive bacterium itself did not seem responsible of this effect. Consequently, this seems to confirm that, through one or several exoproducts or ECVs, *Pseudoalteromonas* sp. 1A1 would be able to prevent eukaryotic cell from caspase-dependent apoptotic cell death and may be to take benefit of it as long as possible prior to the physical contact between protagonists. This scheme is different from what is proposed in the literature (see [40]) where the role of LPS as actors in induced cell death was reviewed. LPS from *Endozoicomonas* sp. Hex311 also did not influence the level of the caspase gene transcription although the alive bacterium as well as it supernatant down-regulated it. *Endozoicomonas* sp. Hex311 LPS would not be apoptogenic while the bacterium or its exoproducts would repress the apoptosis caspase-dependent. *S. domuncula* is accustomed to this symbiotic bacterium and tolerated it within its tissue, not reacting to its presence (within the limits of our study). The study LPS from *Endozoicomonas* sp. Hex311 lead to the same conclusion as for the LPS from the pathogenic *Pseudoalteromonas* sp. 1A1. LPS would not be responsible of the triggering of apoptosis. It is not surprising in light of the down-regulation of the TLR gene in the presence of both bacteria considering the entanglement of the TLR and the apoptosis pathway. Furthermore, alive *Pseudoalteromonas* sp. 1A1 did not influence the expression of this caspase gene, a result in relation to its LPS. It could involve a strategy of emission of chemical messengers adopted by the pathogenic to hide itself from a receptor involved in the immune response and actively modify the sponge cell physiology to its own profit [41].

### 4.4. TRAF-6

The TNF receptor-associated factor-6 has been identified in sponges by Srivastava et al. [42]. As adaptor proteins they link several membrane surface receptors to diverse signaling cascades and regulate cell death or survival through various proteins [43]. *S. domuncula* TRAF-6 gene was down-regulated in the presence of alive *Endozoicomonas* sp. Hex311 and in a stronger manner by its LPS; up-regulated when cell cultures of the sponge were incubated with the supernatant of this bacterium. Alive *Pseudoalteromonas* sp. 1A1 did not affect the expression of TRAF-6 gene while its LPS and its culture supernatant strongly down-regulated it. This may suggest that TRAF-6 may be a relay protein between the *S. domuncula* TLR and further signaling pathways since both the TLR and TRAF-6 genes expression evolved in the same way in the presence of LPS. This is in agreement with some recent results found in the literature concerning mammals [43,44,45] as well as marine invertebrates [46,47,48,49]. Furthermore, concerning the pathogenic bacterium, it seems that TRAF-6 gene expression is mainly affected in response to one/several secreted molecules or vesicles. This suggests at least a partial integration of pathogens perception by different cell surface receptors confirming relevant works described in the literature [43].

### 4.5. MPEG

The macrophage-expressed gene plays a crucial role in innate immunity through membrane attack complex or perforin against pathogen microorganisms [50]. It has been evidenced that LPS [51] regulate the expression of MPEG. The *S. domuncula* perforin-like similar to the MPEG is up-regulated in the presence of Gram-negative bacteria [10]. Whereas LPS from *E. coli* stimulated the expression of the *S. domuncula* MPEG [10], those from *Pseudoalteromonas* sp. 1A1 and *Endozoicomonas* sp. Hex311 did not modify its expression [17]. LPS from *Pseudoalteromonas* sp. 1A1 would not be able to silence the intracellular cell-signaling as mentioned by [27] in order to immunomodulate the antibacterial response. In the present study, we demonstrated that the expression of MPEG was different in the presence of alive bacteria and their secreted molecules. Alive *Endozoicomonas* sp. Hex 311 did not influence the expression of MPEG while its culture supernatant drastically decreased it; alive *Pseudoalteromonas* sp. 1A1 and more obviously its culture supernatant reduced its expression. It is understandable that the bacterium *Endozoicomonas* sp. Hex311 or its LPS did not trigger any antibacterial response since it lives in a long-lasting way in the presence of the eukaryotic cell and, in vitro, requested sponge extract to grow. Surprisingly, one/several secreted molecules or ECV in the culture medium was/were able to trigger an antibacterial response. Nonetheless, note that in the present study, the supernatant of *Endozoicomonas* sp. Hex311 was prepared in vitro, without any contact with the sponge cells. Thus, its composition may be different from that adopted by the bacterium in contact with eukaryote cells leading it to secrete a different panel of molecules. As mentioned above, the pathogenic bacterium *Pseudoalteromonas* sp. 1A1 seems to take advantage of the situation to over-take or by-pass the host’s immune defense for its own profit. This situation is commonly observed among bacterial pathogens and depends on the infected host cells, as for example *Legionella pneumophila* (review for example in [52,53]).

## 5. Conclusions

In the present study, we described at the molecular level the response of an axenic eukaryotic cell challenged with bacteria with different symptomologies, in particular a symbiotic one and a pathogen one. As holobionts [6], sponges co-evolved for billion years with several kinds of bacteria: Some being symbiotic, others opportunistic and pathogens, the last having a role in the diet of the animal. Working with sponges may be suitable to investigate the host response faced by the different bacteria or their exoproducts, and then to understand the establishment and the functioning of the innate immune system of the sister group of all other metazoan known to date, specifically the Porifera [54]. Further studies will need to consider more genes to better decipher how eukaryotic cells in lower metazoan distinguish between mutual, commensal, opportunist and pathogen microorganisms and if it is able to recognize its own cells among all those present in the holobiont.
